# Incidental non-complicated posterior rectus sheath hernia

**DOI:** 10.1259/bjrcr.20190072

**Published:** 2020-02-12

**Authors:** Peter Connell, James Hennebry, Senan Alsanjari, Riddhika Chakravartty, Mona Sabala

**Affiliations:** 1 East Surrey Hospital, Redhill, Surrey, United Kingdom; 2 University of Nottingham, Notingham, United Kingdom

## Abstract

Posterior rectus sheath hernia is a truly rare finding, with only 11 documented cases since the first report in 1937. A posterior rectus sheath hernia is herniation of bowel and/or omentum through the posterior portion of the rectus sheath, but not through any other structure. This can only occur medial to the spigelian fascia, differentiating it from a spigelian hernia. Previous missed cases have led to complications such as bowel incarceration, obstruction or even strangulation and have required surgical intervention. In this case report, we describe an incidental finding of a non-complicated posterior rectus sheath hernia in an 83-year-old male. Annotated cross-sectional imaging provides anatomical context that is not widely available in the existing literature. Due to its rarity and potential complications, it is also important to report this case in order to enhance the evidence base for posterior rectus sheath hernia and to familiarize this uncommon condition to radiologists, clinicians and surgeons.

## Clinical presentation

An 83-year-old male presented with haematuria in November 2018. A CT Urogram was performed for investigation of the haematuria which revealed a small, well-defined, mildly enhancing right renal mass. The outcome from the urology MDT was to have a repeat CT in 6 months. An incidental finding of a left posterior rectus sheath hernia was noted and reported.^1–4^

The patient’s surgical history includes a previous laparoscopic cholecystectomy for cholecystitis in 1991, left inguinal hernia repair in 1992 and a subsequent re-repair with synthetic mesh in 1996. Upon noting the hernia, a retrospective history revealed the patient did experience intermittent abdominal pains. Although the definitive cause cannot be known, it could be related to the hernia.

## Investigations/ Imaging Findings

**Figure 1.**
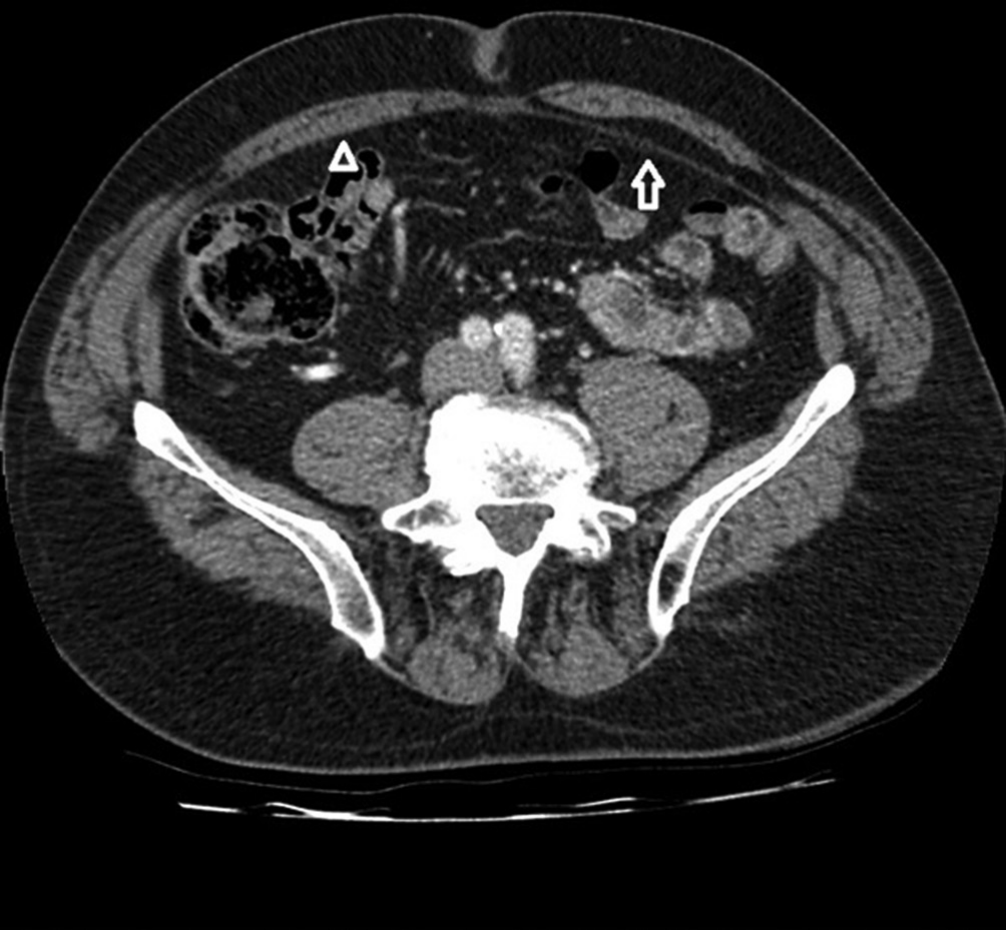
A contrast-enhanced, portal venous phase axial CT image shows splitting of the posterior sheath on the left side (white arrow) with intact sheath on the right side (arrowhead).

**Figure 2.**
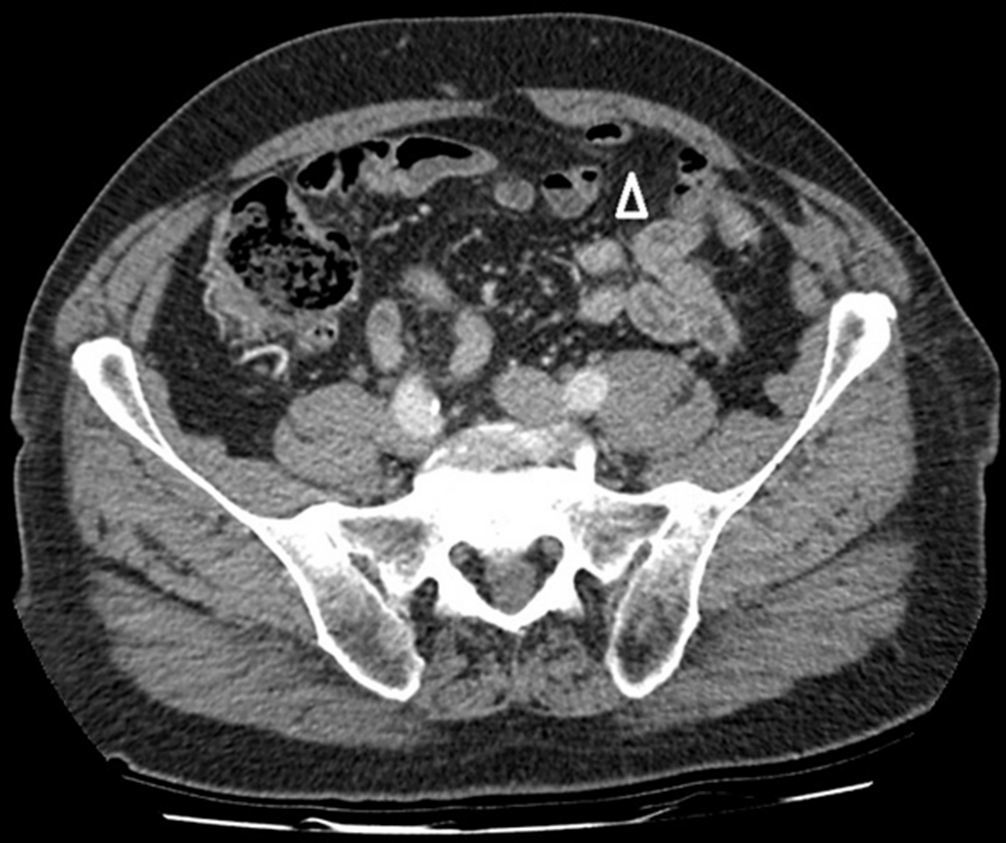
A contrast-enhanced, portal venous phase axial CT image at a lower level reveals the defect (arrowhead) which is located in the infraumbilical region, below the arcuate line.

**Figure 3.**
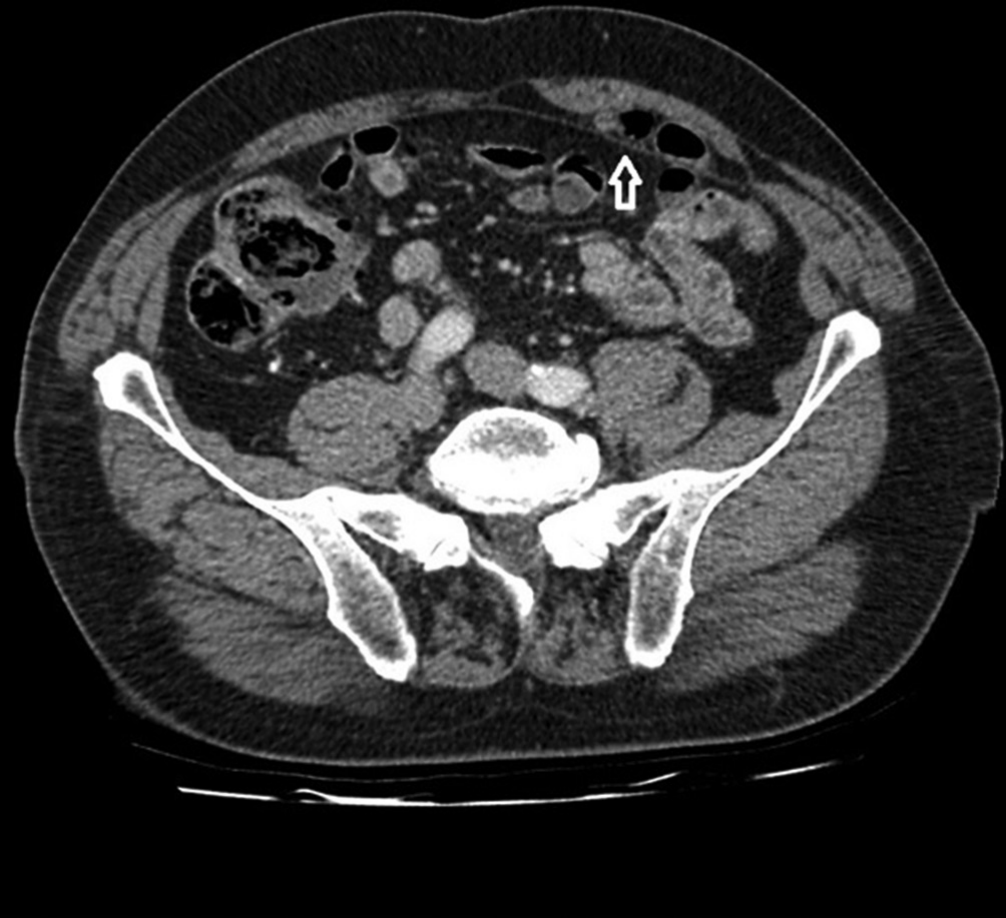
An additional contrast-enhanced, portal venous phase axial CT demonstrates the herniated bowel loops (arrow). The loops are not dilated and hence there is no bowel obstruction.

**Figure 4.**
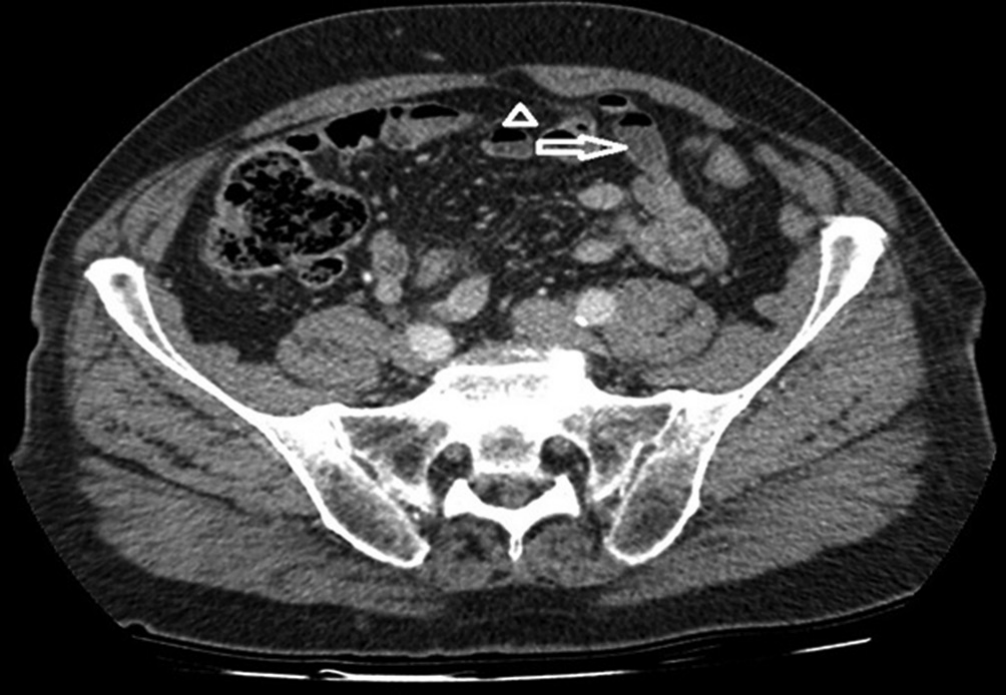
Another contrast-enhanced portal venous phase axial CT image again shows the split sheath (arrowhead) and a bowel loop entering the defect (arrow).

**Figure 5.**
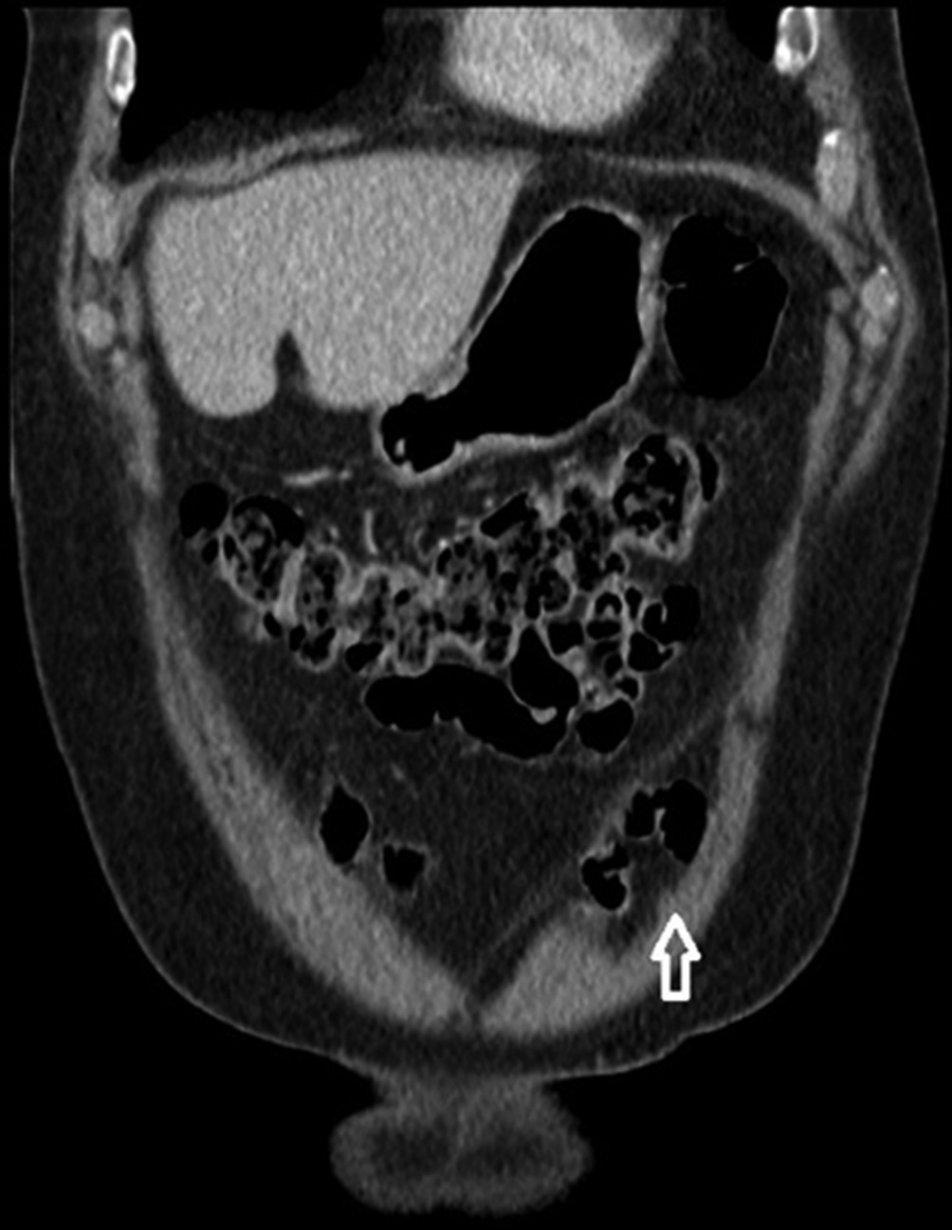
A contrast-enhanced portal venous phase coronal reformat reveals the hernial sac (arrow). Note that the hernia does not exit into the subcutaneous tissues.

**Figure 6.**
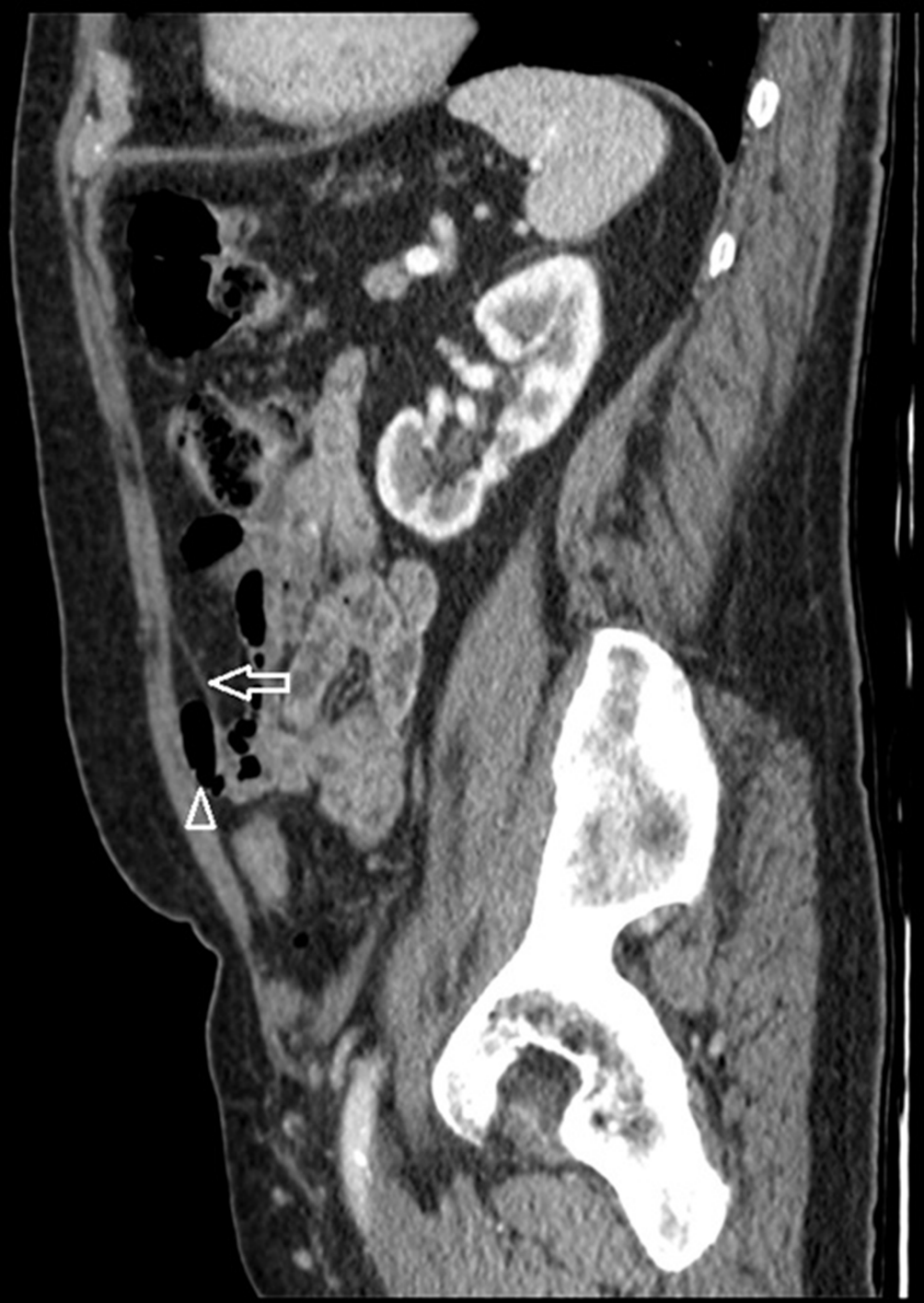
A contrast-enhanced portal venous phase sagittal reformat shows bowel loops (arrowhead) within the space between the rectus abdominis muscle anteriorly and the separated rectus sheath posteriorly (arrow).

A CT Urogram and a 6 month follow-up Triple Phase CT of the Kidneys clearly demonstrated the posterior rectus sheath hernia with almost no significant interval changes. The contrast enhanced, portal venous phase CT imaging shows splitting of the posterior sheath of the left rectus abdominis muscle with intact posterior sheath on the right side (Figure 1). The defect in this case is not large, measuring about 2 cm and is typically located in the infraumbilical region, below the arcuate line, in contrast to the majority of previously reported cases where the defect occurred in the supraumbilical region (Figure 2). Multiple small bowel loops are herniated through the defect, yet there is no evidence of bowel obstruction (Figures 3 and 4). Contrast-enhanced, portal venous phase coronal and sagittal reformats were also obtained which provide more detail (Figures 5 and 6).

## Treatment and prognosis

Due to the obscurity of the pathology, specific management does not have a good evidence base or NICE guidance. As such, the management has been based upon the general principles of hernia management.^5^ Previous cases have been managed conservatively, with surgical repair only occurring if the hernia becomes significantly symptomatic or strangulates. In this case, the patient has no significant symptoms and the appearances are stable on imaging. Therefore, conservative approaches have been taken.

Potential complications documented in the literature are comparable for other hernias, the most significant of which is ischaemia of the bowel.^4^

## Outcome

No specific follow-up plan has been arranged given the incidental and largely asymptomatic nature of the hernia. The patient did have follow-up imaging for a renal mass which showed stable appearances of the hernia. Any further interventions should be considered when and if the hernia becomes symptomatic.

## Differential diagnosis

The main differential diagnosis is a spigelian hernia. This can be differentiated from a posterior rectus sheath hernia as a spigelian hernia occurs lateral to the rectus abdominis muscle and typically bulges into the subcutaneous tissue. A posterior rectus sheath hernia does not bulge into the subcutaneous tissues and occurs posterior to the rectus abdominis muscle.^6^

Posterior rectus sheath hernia can also present with bowel obstruction. In this context, the differentials would include other causes of bowel obstruction, for example; adhesions relating to previous surgery, malignancy, pseudo-obstruction or ileus.^3^

## Discussion

The rectus sheath is a tough fibrous compartment which contains the rectus abdominis muscle and pyramidalis muscle. It extends from the inferior costal margin and ribs 5, 6 and 7 to the crest of the pubis. The sheath has anterior and posterior layers that fuse laterally at the linea semilunaris and in the midline at the linea alba. Above the arcuate line, the anterior compartment consists of the fascia from the external oblique and half of the internal oblique. The posterior compartment is made up from the fascia of the other half of internal oblique, the fascia of transversus abdominis and the transversalis fascia. Below the arcuate line, the anterior compartment is made of fascia from all three lateral abdominal wall muscles and only the transversalis fascia contributes to the posterior compartment^2^ making it weaker and more susceptible to herniation.

Posterior rectus sheath hernia is an exceedingly rare type of anterior abdominal wall hernias with only scarce cases recorded. It also belongs to the subtype of interparietal hernias in which the hernial sac unusually lies between the various layers of the abdominal wall muscles and does not exit into the subcutaneous tissue as other abdominal wall hernias do.

The exact cause of these hernias is still not clear with the limited literature available suggesting three main causes: congenital; post-surgical and traumatic (Lenobel, Lenobel and Yu, 2014).^3^ Considering this patient’s previous history of hernias, it could be that there is either a congenital propensity to hernias or the hernia has occurred because of previous surgical interventions. In addition, similar to other types of hernias, any conditions which increase intra-abdominal pressure such as obesity, ascites and progressive muscle weakness increase the likelihood of these hernias.

Posterior rectus sheath hernia diagnoses have historically been proven by surgery. CT scanning appears to be a modality with high sensitivity for the condition allowing easy and accurate diagnosis, as those cases diagnosed on CT have all been confirmed on surgical exploration.^4^ Some cases have demonstrated recurrence and complications, highlighting the importance of recognizing it in asymptomatic patients.^3^

The management of posterior rectus sheath hernias is currently based upon the general principles of hernia management. Recent evidence has emerged that highlights the potential role of laparoscopic repair in selected cases of abdominal wall hernias compared with traditional open techniques.^7^

In conclusion, this case describes a non-complicated posterior rectus sheath hernia with labelled cross-sectional imaging that will act as an aid for radiologists and surgeons in identifying future cases. A small but growing body of cases are now available, including those which focus on surgical techniques. Further research in this area is required to standardize management.

## Learning points

- A posterior rectus sheath herniation is rare but important to recognize and can be easily overlooked.
- A lack of familiarity with the nonspecific presentation may result in delayed or even misdiagnosis of this type of hernia.
- The recent availability and the widespread use of cross-sectional imaging in diagnosis has added a superior and detailed anatomical view sufficient enough to allow easier diagnosis and classification of herniation.
